# The Effect of Continuing Chemotherapy after Chemoradiotherapy during the Time to Surgery on Tumor Response and Survival for Local Advanced Rectal Cancer

**DOI:** 10.1155/2022/4108677

**Published:** 2022-09-15

**Authors:** Atike Gökçen Demiray, Arzu Yaren, Uğur Sungurtekin, Papatya Bahar Baltalarlı, Neşe Demirkan, Duygu Herek, Burcu Yapar Taşköylü, Gamze Gököz Doğu, Serkan Değirmencioğlu, Utku Özgen, Halil Sağınç, Umut Çakıroğlu, Nail Özhan, Canan Karan, Burçin Çakan Demirel, Tolga Doğan, Melek Özdemir

**Affiliations:** ^1^Department of Medical Oncology, Pamukkale University Faculty of Medicine, Denizli-, Turkey; ^2^Department of Surgery, Pamukkale University Faculty of Medicine, Denizli-, Turkey; ^3^Department of Radiation Oncology, Pamukkale University Faculty of Medicine, Denizli-, Turkey; ^4^Department of Medıcal Pathology, Pamukkale University Faculty of Medicine, Denizli-, Turkey; ^5^Department of Radiology, Pamukkale University Faculty of Medicine, Denizli-, Turkey

## Abstract

**Aim:**

The current standard treatment of locally advanced rectal carcinoma is total mesorectal excision and postoperative adjuvant chemotherapy after neoadjuvant concurrent chemoradiotherapy (NCRT). Many studies have shown that pathological complete response (pCR) is an important prognostic factor for patients receiving NCRT. Many studies have therefore been conducted to increase pCR rates by changing the perioperative treatment strategies. Prolonging the chemotherapy time may be a reasonable way to increase the effectiveness of NCRT, pCR, and survival rates. We investigated whether neoadjuvant consolidation chemotherapy had an effect on tumor response and survival.

**Methods:**

The data of 163 patients diagnosed with locally advanced rectal carcinoma were evaluated. The data of 107 patients (Group 1) who were radiologically T3–T4 and/or N+ and received chemotherapy after NCRT until their operations were compared with the data of 56 patients (Group 2) who were operated after NCRT.

**Results:**

Group 1 patients had tumor and node downstaging. Their pCR was found significantly higher than in Group 2 (*p* = 0.005). In Group 1 patients with T3, pCR was significantly higher than for those with T4. The elapsed time between NCRT and surgery was significantly longer in patients with pCR (respectively, *p* = 0.012 and *p* = 0.008).

**Conclusion:**

Neoadjuvant consolidation chemotherapy after NCRT is a safe approach that can lead to higher pathological complete response rates. The time until surgery with neoadjuvant consolidation chemotherapy may provide the chance to follow the patient without surgery in addition to increasing pCR.

## 1. Introduction

The current standard treatment of locally advanced rectal carcinoma is total mesorectal excision (TME) and postoperative adjuvant chemotherapy after neoadjuvant concurrent chemoradiotherapy (NCRT), which includes a multimodal approach. Local recurrence rates range from 25% to 50% for patients with T3–T4 and/or node-positive cancer [1]. The risk of local recurrence is significantly reduced with NCRT [2]. However, the survival benefit of NCRT has not been demonstrated, and the rate of distant metastasis is still reported to be as high as 30% [3]. Since systemic control is not as good as local control (possibly due to insufficient control of micrometastases), this has led to the search for new alternative strategies in neoadjuvant therapy for distant metastasis control and higher chances of survival.

Increased survival rates have been observed in patients with pathological complete response (pCR) after NCRT, and many studies have shown that pCR is an important prognostic factor for rectal cancer patients receiving NCRT. In these studies, the rate of pCR was reported to be 15% to 30% [4, 5]. Many studies have therefore been conducted to increase pCR rates by changing the perioperative treatment strategies. Many researchers have tried various chemotherapeutic regimens to increase the rate of pCR, resulting in improved survival rates. These efforts were based on the results of adding chemotherapy to neoadjuvant radiotherapy (RT), which enhances the radiation effect as a radio sensitizer. However, the antitumor effectiveness of NCRT in particular remains unclear. Until now, there has been no single chemotherapeutic agent that has significantly enhanced the pathological complete response.

Prolonging the chemotherapy time may be a reasonable way to increase the effectiveness of NCRT, the rate of pCR and the chances of survival. After the completion of conventional NCRT therapy, there is a six-to-eight-week rest period until surgery to achieve tumor response to concurrent chemoradiotherapy (CRT) and recovery from CRT-related toxicity. Habr-Gama et al. reported a 65% clinical complete response rate after administering the same chemotherapy for three additional cycles during the rest period following the completion of six-week 5-fluorouracil (5-FU)-based CRT. They thought that additional chemotherapy could increase the pCR rate with both a radio-sensitizing effect and a potential antitumor effect [6]. In this case, an alternative approach would be to apply a safe, effective, low-toxicity, and low-cost chemotherapy regimen during the ‘rest' period after CRT is completed for rectal cancer patients receiving neoadjuvant therapy. This approach will allow for optimal radio-sensitizing effects from chemotherapy and will deliver the potential antitumor effects of chemotherapy at an effective systemic dose. With this method, it is predicted that there may be regression in tumor size and nodal involvement as well as an increase in pathological complete response rates and survival rates. In our study, we investigated whether additional chemotherapy was effective towards tumor response and survival rates in the period before surgery.

## 2. Method

### 2.1. Patients

The data of 163 patients diagnosed with locally advanced rectal carcinoma who were followed up in the Department of Medical Oncology, Faculty of Medicine, at Pamukkale University were evaluated. The data of 107 patients (Group 1) who were radiologically T3–T4 and/or N+ and who received chemotherapy after undergoing neoadjuvant CRT up to their operations were compared with the data of 56 patients (Group 2) who were operated on after undergoing neoadjuvant CRT. The patients received long-term radiotherapy (50.4 Gy for 6 weeks) and RT with capecitabine (825 mg/m2, orally, twice daily 5 days/week+CRT×5 weeks) or 5-FU (225 mg/m2, intravenous over 24 hours 5 days/week during CRT) in neoadjuvant CRT. Group 1 patients were given capecitabine (1000 mg/m^2^ PO twice daily for 14 days every 3 weeks), XELOX (oxaliplatin 130 mg/m^2^ intravenous day 1, capecitabine 1000 mg/m^2^ PO twice daily for 14 days every 3 weeks), FOLFOX (oxaliplatin 85 mg/m^2^ IV, day 1, leucovorin 400 mg/m^2^ IV day 1,^b^5-FU 400 mg/m^2^ IV bolus on day 1, followed by 1200 mg/m^2^/day×2 days (total 2400 mg/m^2^ over 46 hours) continuous infusion, repeat every 2 weeks), or De Gramont (Leucovorin 200 mg/m^2^ 2 hour infusion, bolus injection of 5-FU 400 mg/m^2^ then, 22-hour infusion of 5-FU 600 mg/m^2^ for 4 day, repeat every 2 weeks) between NCRT and surgery. After the operation, the patients were given adjuvant chemotherapy. The extent of the primary tumor was assessed by Magnetic Resonance Imaging (MRI) in all patients. High-resolution, T2-weighted MRI was performed before neoadjuvant treatment and surgery. The clinical staging (tumor and node involvement) of the patients before neoadjuvant treatment and preoperative evaluation of the response to treatment was evaluated by MRI.

### 2.2. Ethics approval

This study was approved by the ethics committee of Pamukkale University, School of Medicine (Approval number: 60116787-020/36932, approval date: 07.06.2017).

### 2.3. Statistical Analysis

The categorical descriptive statistics were performed using the chi-squared (*x*^2^) test. Survival analysis was shown in Kaplan-Meier curves and analyzed using log-rank tests and multivariate Cox proportional hazards models. All statistical analyses were performed using the Statistical Package for the Social Sciences (SPSS) 23.0. (IBM Corp.; Armonk, NY, USA). A 2-sided *p* < 0.05 was considered statistically significant.

## 3. Results

Of the 163 patients with locally advanced rectal carcinoma, 107 were Group 1 patients and 56 were Group 2 patients. The demographic and clinical characteristics of Group 1 and Group 2 patients are summarized in Table 1. All the patients had an ECOG performance of ≤1 and had normal bone marrow, liver, and kidney functions. Ninety-six patients were operated on and postoperative pathological staging was implemented. The Group 1 patients were given capecitabine, XELOX, FOLFOX, or De Gramont a during the rest period (Table 2). No toxicity was observed during these chemotherapies.

Downstaging between the preoperative clinical stages and the postoperative pathological stages in Group 1 was shown in Table 3. Group 1 patients had tumor downstaging and node downstaging and also had no lymphovascular invasion. Their complete pathological response was found to be significantly higher than in Group 2 (Table 4).

In Group 1 patients with T3, the complete pathological response was significantly higher than for those with T4. The elapsed time between neoadjuvant CRT and surgery was significantly longer in patients with pCR (Table 5).

Local recurrences were detected within the first two years after the operation. Recurrence was detected in 26 patients in Group 1. Local metastasis was found in three of these patients, distant metastasis in 22 and both local and distant metastasis in one.

The mean survival of the whole group (Group 1 + Group 2) was 66.9 months. Group 1 survived for 70.6 months, while Group 2 survived for 50 months. The difference, however, was not statistically significant (*p* = 0.242). The 3- and 5-year survival rates in Group 1 were 74% and 59%, respectively. In Group 2, the 3- and 5-year survival rates were 64% and 28%, respectively. Regarding the mode of operation, the mean length of survival was significantly longer in the LAR group compared to the APR group (69.91 months and 48.8 months, respectively; *p* = 0.032). The mean length of survival of the group who received postoperative adjuvant chemotherapy was significantly longer than for those who did not (68.9 months and 41.3 months, respectively; *p* = 0.041).

The mean relapse time for the whole group was 72.4 months. While the mean of Group 1 was 68.8 months, the mean of Group 2 was 57.7 months. However, the difference was not statistically significant (*p* = 0.304). When the rectum parts were examined, the time to recurrence was significantly longer in the middle rectum, in those with tumor downstaging and in those with concurrent CRT (*p* = 0.011, *p* = 0.024, and *p* = 0.039, respectively).

The mean survival time of patients with pCR in the whole group was 83.7 months, while the mean survival time of patients without pCR was 65.5 months. The difference was statistically significant (*p* : 0.048). In Group 1 patients, 2 patients with pCR relapsed and 2 patients died. In group 1 patients, the mean survival time was 83.2 months and 70.2 months in patients with and without complete pathological response, respectively, while the time to relapse was 32.5 months and 19.8 months, respectively. However, these differences were not statistically significant (*p* = 0.159 and *p* = 0.580, respectively). With multivariate analysis, no parameter that significantly affected the time to recurrence and survival was found.

## 4. Discussion

We think that our study is important because additional chemotherapy increases pCR significantly in the period until surgery in patients with locally advanced rectal cancer, and may provide the opportunity to follow this group of patients without surgery in the future, in terms of showing real-life data. We hope that it can shed light on other studies to be conducted in this respect. In a nonrandomized phase-2 study in which Garcia-Aguilar et al. investigated the efficacy of consolidation chemotherapy between neoadjuvant CRT and TMJ in patients with locally advanced rectal cancer, patients modified two, four, or six cycles between neoadjuvant CRT and resection at different interval times of 12, 16, and 20 weeks. They bought FOLFOX. The pCR of patients who received consolidation chemotherapy resulted in an improvement of 25%, 30%, and 38%, respectively, compared with 18% for patients without consolidation chemotherapy. In our study, while pCR was 20.6% in patients who received additional chemotherapy until surgery, pCR was 5.4% in patients who did not receive chemotherapy during this period [7]. In a retrospective cohort study of Cui et al. demonstrating the efficacy of consolidation chemotherapy (between neoadjuvant CRT and operation) in patients with locally advanced rectal cancer, they showed increased pCR responses. In this study, 63 patients received 2 cycles of XELOX consolidation chemotherapy; it was not given to 61 patients. Looking at the results, they had similar rates with our study. While pCR was 19.3% in the group that received consolidation, it was 4.9% in the group that did not. In this study, tumor downstage was 45.8% in the group that received consolidation and 24.6% in the group that did not [8]. In our study, both T and N downstage were significantly higher in the group that received consolidation. Patients who received consolidation chemotherapy and had a complete pathological response had a longer period (6-10 weeks) until the operation after neoadjuvant CRT. In our study, this period was significantly longer in patients with pathological complete response who received consolidation chemotherapy than those who did not receive consolidation chemotherapy. In this study, it was not specified whether consolidation chemotherapy or the length of time until the operation was effective in the pathological complete response. In our study, both significantly affect pCR. However, no significant difference in efficacy on pCR was found between consolidation chemotherapy protocols and the number of cycles.

In a randomized phase-2 study conducted by Kim et al., consolidation chemotherapy showed increased pCR rates and tumor downstaging. In this study, 53 patients received 2 courses of XELOX consolidation chemotherapy and 55 patients were not. While the rate of pCR was 13.6% in the group receiving consolidation chemotherapy, it was 5.8% in the group not receiving consolidation chemotherapy; tumor downstage was 36.4% and 21.2%, respectively [9]. In a prospective, phase-2 Korean study, patients were given capecitabine for a further 4 weeks during the rest period until the operation after 6 weeks of concurrent RT with capecitabine. The rate of pCR, T and N downstage in these patients was similar to our study (20.9%, 74.4%, and 76.7%, respectively) [10].

The efficacy of total neoadjuvant therapy was investigated in a prospective Slovenian study in patients with high risk locally advanced rectal cancer. As total neoadjuvant therapy, 4 cycles of induction XELOX/FOLFOX followed by simultaneous radiotherapy with capecitabine and 2 cycles of XELOX/FOLFOX chemotherapy were administered until the operation. 66 patients were included in the study, 61 patients received induction chemotherapy. All patients received concomitant CRT, and 54 patients received consolidation chemotherapy. As a result, the pCR rate was 23.3% in 60 patients who were operated [11]. Only grade 1 and 2 toxicity were observed due to intensive treatment. The median time from the concurrent CRT end to the operation was 11 weeks, which was 11 weeks in our study. Although a similar protocol was not used in our study, the number of patients in the group that received consolidation was higher and we had a control group. We had a rate of pCR close to an intensive treatment such as total neoadjuvant therapy, which may have a higher toxicity risk. Therefore, prospective, randomized studies that can compare multiple arms are needed. In a multicenter phase-2 study, 211 patients were nonrandomized into 4 branches, who were not receiving consolidation mFOLFOX chemotherapy after neoadjuvant CRT and receiving 2-4-6 cycles of consolidation mFOLFOX chemotherapy. Pathological complete response rate in the whole group was 27.5%. [12] This rate was 16.4% in our study. However, this rate increased to 20.6% in the group that received consolidation chemotherapy. Although the disease-free survival rate was significantly lower in patients not receiving consolidation chemotherapy in this study, there was no difference between the groups in terms of mean survival. However, disease-free survival and mean survival rate were significantly higher in patients with pathological complete response. In our study, no significant difference was found in terms of time to relapse and survival. These differences may not have arisen due to the fact that there is no equal distribution between chemotherapy protocols and they take different and few cycles.

Low acute toxicity was found in the SCRT arm in a study comparing oxaliplatin and concurrent CRT with preoperative 3 cycles of FOLFOX chemotherapy after SCRT. No difference was found between the rates of local control, R0 resection, and distant metastasis in both arms. However, survival was significantly higher in the arm in SCRT [13]. The effect of short-term radiotherapy (25 Gy in one week) and long-term chemoradiotherapy (5-6 weeks, concurrent with chemotherapy and more than 45 Gy in total) on local control have been the subject of many studies. In two randomized controlled studies comparing these, no difference was found between local control and surgical procedure. However, pCR rates were significantly better in the CRT arm [14, 15]. The absence of downstage after SCRT in these two studies can be explained by the short time between SCRT and surgery. Several other studies have shown what causes tumor downstage when the interval between SCRT and surgery is extended [16–18]. In the Stockholm III study, the longer time until SCRT and surgery was found to be more effective in tumor downstaging than only long-term radiotherapy [19]. Therefore, it was emphasized that there should be no doubt for the local effect of SCRT, especially with the addition of chemotherapy. Surgery in the SCRT arm was performed 4-8 weeks after the end of RT. This long-term radiotherapy or SCRT did not cause low local control and more postop complications compared to the immediate operation arm [20].

In a study investigating whether neoadjuvant intensified therapy improves overall survival and disease-free survival in locally advanced rectal cancer, it was shown that adding oxaliplatin to standard therapy (5-FU) had a beneficial effect on survival by increasing pathological complete response and local control. In addition, it was well tolerated. This study showed that intensified treatment strategies in the neoadjuvant period positively affect tumor response. However, it is not yet clear when these treatment strategies should be given (concurrent with radiotherapy, after radiotherapy or continuously until surgery?) and which agents should be given for how long. New treatment strategies are necessary [21].

In the Phase-3 RAPIDO study, the arm of short-term radiotherapy (5×5 Gy, week, SCRT) followed by preoperative chemotherapy followed by standard versus operation (long-term simultaneous CRT followed by operation) was compared. Adjuvant chemotherapy was given in both arms. In the study, the tolerability and compliance of the research arm were evaluated in terms of surgical procedures and postoperative complications. When the two groups were compared, preoperative chemotherapy compliance rate in the study arm was found to be higher than postoperative chemotherapy. Grade 3 oxaliplatin toxicity rate was higher in the research arm. However, these resection rates, surgical procedures, and postoperative complications did not change, and no significant difference was found in this regard in both arms. RAPIDO trial showed that short-course radiotherapy followed by 18 weeks of chemotherapy before surgery decreases the probability of disease-related treatment failure compared with chemoradiotherapy with or without adjuvant chemotherapy. Additionally, the high rate of pathological complete response can potentially contribute to organ preservation. Supported by high compliance and tolerability, this treatment could be considered as a new standard for high-risk locally advanced rectal cancer. Future research could focus on assessing tumor response to preoperative treatment at an early stage and improving the efficacy of systemic therapy [22].

In the Phase-3 PRODIGE-23 study, patients were randomized to either the neoadjuvant chemotherapy group or standard-of-care group. The neoadjuvant chemotherapy group received neoadjuvant chemotherapy with FOLFIRINOX (oxaliplatin 85 mg/m^2^, irinotecan 180 mg/m^2^, leucovorin 400 mg/m^2^, and fluorouracil 2400 mg/m^2^ intravenously every 14 days for 6 cycles), chemoradiotherapy (50 Gy during 5 weeks and 800 mg/m^2^ concurrent oral capecitabine twice daily for 5 days per week), total mesorectal excision, and adjuvant chemotherapy (3 months of modified FOLFOX6 (intravenous oxaliplatin 85 mg/m^2^ and leucovorin 400 mg/m^2^, followed by intravenous 400 mg/m^2^ fluorouracil bolus, and then, continuous infusion at a dose of 2400 mg/m^2^ over 46 h every 14 days for six cycles) or capecitabine (1250 mg/m^2^ orally twice daily on days 1–14 every 21 days)). The standard-of-care group received chemoradiotherapy, total mesorectal excision, and adjuvant chemotherapy (for 6 months). Disease-free survival and metastasis free survival were significantly longer in patients treated with neoadjuvant FOLFIRINOX. Induction therapy with six cycles of FOLFIRINOX did not compromise radiotherapy compliance or surgical quality and did not increase local relapses. Neoadjuvant FOLFIRINOX showed significant improvements in the pathological complete response rate compared with standard chemoradiotherapy. Intensification of chemotherapy using FOLFIRINOX before preoperative chemoradiotherapy significantly improved outcomes. The significantly improved disease-free survival in the neoadjuvant chemotherapy group that the perioperative approach is more efficient and better tolerated than adjuvant chemotherapy. [23] RAPIDO and PRODİGE-23 studies showed that multimodal strategies meaning to intensify preoperative treatment by delivering both radiotherapy and systemic dose chemotherapy improved outcomes in patients with locally advanced rectal cancer. The selection of patients who will most benefit from intensification of neoadjuvant therapy and the selection of the most appropriate total neoadjuvant therapy to adopt are still unclear [24].

There are some limitations of this study which may have an impact on the outcomes. These include as follows: (1) low number of patients and (2) consolidation chemotherapy protocols and numbers were different.

In conclusion, our study showed that the addition of neoadjuvant consolidation chemotherapy after CRT is a safe approach that can lead to higher pCR rates, increased compliance with systemic chemotherapy regimens, and longer survival, although not significantly. The time until surgery with neoadjuvant consolidation chemotherapy may provide the chance to follow the patient without surgery in addition to increasing the pCR. For this purpose, randomized prospective studies with a large number of patients are needed.

## Figures and Tables

**Table 1:** Demographic and clinical characteristics of Group 1 and Group 2 patients.

		Group 1(*n*, %)	Group 2 (*n*, %)
Age (median)		61	65
Gender	Male	66 (61.7%)	38 (67.9%)
	Female	41 (38.3%)	18 (32.1%)
Tumor	T3	47 (43.9%)	24 (42.9%)
	T4	60 (56.1%)	32 (57.1%)
Node involment	Negative	29 (27.1%)	33 (58.9%)
	Positive	78 (72.9%)	23 (41.1%)
Rectum	Lower	41(38.3%)	27(48.2%)
	Middle	58(54.2%)	20(35.7%)
	Upper	8(7.5%)	9(16.1%)
Concurrent CRT^∗^	+	94 (87.9%)	49(87.5%)
	-	13 (12.1%)	7(12.5%)
Time between neoadjuvant CRT and surgery (median-month)		10	7
Concurrent CT∗∗	Capecitabine	72(67.3%)	9(16.1%)
	5-fluorouracil	22 (20.6%)	40(71.4%)
	-	13(12.1%)	7(12.5%)
Surgery	-	4(4%)	0 (0%)
	LAR^1^	80(80%)	45(80.4%)
	APR^2^	16(16%)	11(19.6%)
Dissected lymph nodes (median)		5	10
Adjuvant CT∗∗	+	76(71%)	56 (100%)
	-	31(29%)	0(0%)
Relapse	-	80(75.7%)	46 (82.1%)
	+	26 (24.3%)	10(17.9%)
Relapse	Local	3 (2.8%)	2(3.6%)
	Distant	22(20.6%)	8 (14.3%)
	Local+distant	1(0.9%)	0(0%)
Survive	Live	80(74.8%)	37(66.1%)
	Exitus	27(25.2%)	19(33.9%)

^∗^CRT: Chemoradiotherapy; ^∗∗^CT: Chemotherapy; ^1^LAR: Low Anterior Resection; ^2^APR: Abdominoperineal Resection.

**Table 2:** Chemotherapy protocols and number of cycles given in Group 1.

Number of cycles	Chemotherapy protocols			
	Capecitabine(n)	Xelox(n)	Folfox(n)	De-Gramont(n)
1	0	5	5	0
2	1	16	17	3
3	0	52	3	1
4	0	1	0	1
5	0	1	0	0

**Table 3:** Downstaging between preoperative clinical stages and postoperative pathological stages in Group 1.

Preoperative clinical stage	Postoperative pathological stage						
	pCR∗	T1	T2	T3	T4	Node (-)	Node (+)
T3	15	1	15	8	0		
T4	7	1	16	24	5		
Node (-)	7					27	2
Node (+)	15					45	22

^∗^pCR: Complete pathological response.

**Table 4:** Treatment responses and pathological characteristics of Group 1 and Group 2 patients.

		Group 1 (*n*, %)	Group 2 (*n*, %)	*p*
*T* downstage	-	13(13.5%)	16(28.5%)	**0.023**
	+	83(86.5%)	40(71.4%)	
Node downstage	-	51(47.7%)	46(82.1%)	**0.001**
	+	45(46.9%)	10(17.9%)	
Lymphovascular invasion	-	87(90.6%)	41(73.2%)	**0.005**
	+	9(9.4%)	15(26.8%)	
Perineural invasion	-	83(86.5%)	47(83.9%)	0.669
	+	13(13.5%)	9(16.1%)	
	-	74 (69.2%)	53 (94.6%)	**0.005**
Pathological complete response	+	22 (20.6%)	3 (5.4%)	

**Table 5:** Comparison of the characteristics of Group 1 patients with and without pathological complete response.

		Pathological response		*p*
		Complete pathological response (pCR)(*n*)	Nonpathological complete response (non-pCR)(*n*)	
Tumor	T3	15	28	**0.012**
	T4	7	46	
Node	Negative	7	22	0.851
	Positive	15	52	
Rectum	Lower	8	27	0.727
	Middle	13	40	
	Upper	1	7	
Concurrent CRT∗	Capecitabine	16	48	0.507
	5-fluorouracil	5	16	
	-	1	10	
Time between neoadjuvant CRT∗ and surgery (median-month)		11	9	**0.008**
Chemotherapy protocols	Capecitabine	0	1	0.281
	Xelox	19	49	
	Folfox	3	19	
	De-Gramont	0	5	
Relapse	-	20	54	0.079
	+	2	20	
Relapse	-	20	54	0.203
	Local	0	2	
	Distant	2	18	
Surgery	LAR^1^	20	60	0.277
	APR^2^	2	14	
Node downstage	-	7	44	0.023
	+	15	30	
*T* downstage	-	0	13	0.034
	+	22	61	
Survive	Live	20	54	0.079
	Exitus	2	20	

## Data Availability

The data used to support the findings of this study are included within the article.

## Additional Points

*What does this paper add to the literature*? This is the first clinical study from Turkey showing that consolidation chemotherapy and a long waiting time for surgery increase the pathological complete response. Although these results have not yet statistically increased survival, they may support the chance of these patients being followed up without surgery in the future.
